# The tumor coagulome as a potential biological determinant of postsurgical recurrence of oral squamous cell carcinoma

**DOI:** 10.3389/froh.2025.1554739

**Published:** 2025-02-24

**Authors:** Zuzana Saidak, Antoine Galmiche

**Affiliations:** ^1^ UR7516 CHIMERE, Université de Picardie Jules Verne, Amiens, France; ^2^ Service de Biochimie, Centre de Biologie Humaine (CBH), CHU Amiens Picardie, Amiens, France

**Keywords:** oral squamous cell carcinoma, postsurgical recurrence, coagulome, tumor microenvironment, transcriptomics

## Abstract

**Objectives:**

The tumor coagulome is an intrinsic characteristic of human tumors and a key determinant of cancer-associated thrombosis (CAT). Oral Squamous Cell Carcinoma (OSCC) establish a local procoagulant state that contributes to a broad range of vascular complications, and potentially also to tumor progression. Recent clinical studies suggest that biomarkers of coagulation might be of interest for predicting postsurgical recurrence of OSCC, but it remains unclear whether specific properties of the coagulome of OSCC are conducive to postsurgical recurrence. We examined this possibility using transcriptomic analyses of OSCC.

**Materials and methods:**

Using bulk RNA-seq data from TCGA and other sources, we explored the link between the coagulome (*n* = 85 genes) and disease-free survival (DFS) of OSCC with machine-learning. Tumor microenvironment analyses and single-cell RNA-seq analyses were used to address the potential mechanisms that link coagulation and tumor recurrence. We also compared the coagulome of matched primary/recurrent OSCC.

**Results:**

We identified seven coagulation-related genes, either positively (*F3, F2, F8* and *PROC*) or negatively (*VWF, SERPING1, BDKRB2*) linked to postsurgical recurrence in OSCC at low/intermediate risk, and we validated the model in an independent cohort. We examined their relationship with the tumor microenvironment, suggesting tumor infiltration by T cells as an element of mechanistic explanation. Increased expression of procoagulant genes, such as *F3*, was noted in recurrent compared to matched primary OSCC.

**Conclusion:**

Our observations suggest that active coagulation shapes the oncological outcome of surgery. Analyzing the tumor procoagulant status might help predict postsurgical recurrence of OSCC.

## 1 Introduction

Surgical resection, often combined with adjuvant therapy, is the cornerstone of the treatment of locally-advanced Oral Squamous Cell Carcinoma (OSCC) (1, 2). Despite the progress of the last decade, local tumor recurrence remains frequent and constitutes a major challenge. A better understanding of the biological characteristics of tumors that underlie recurrence remains an unmet need. This especially applies to the tumors considered to be at low/intermediate risk, i.e., those without strong positive predictors of tumor recurrence, such as extracapsular spread or positive surgical margins following resection (1, 2). A better understanding of the biological mechanisms that account for tumor recurrence would help improve medical practice by identifying new biomarkers (3, 4).

Tumor cells express key regulators of the coagulation and fibrinolysis cascades. The tumor coagulome encompasses the multiple genes and proteins that collectively regulate the local equilibrium between coagulation and fibrinolysis, and it represents an intrinsic characteristic of tumor biology (5, 6). The expression of Tissue Factor (TF, encoded by *F3*) has attracted considerable attention as an essential determinant of cancer-associated thrombosis (CAT) (7, 8). CAT is known to be a significant source of mortality and morbidity in cancer patients, accounting for venous thromboembolic events (VTE) and several serious complications, such as pulmonary embolism (9). Through recent pan-cancer studies, OSCC were found to be the tumor type with the highest expression of *F3*, yet with great individual heterogeneity (10, 11). In the surgical context, a systemic activation of the coagulation cascade occurs as a consequence of surgical bleeding, blood stasis induced by vessel clamping, and endothelial injury/inflammatory response initiated by surgical trauma. This systemic activation of coagulation might enhance the local procoagulant response. Prospective studies reveal that in the absence of thromboprophylaxis, up to13% of patients undergoing surgery for head and neck cancers experience VTE and its potentially life-threatening complications (12).

The consequences of the activation of the coagulation cascade extend beyond VTE. The formation of a fibrin polymer, recently compared to a nest formed around cancer cells, could confer specific biological properties to the tumor tissue (13). Coagulation proteases, such as thrombin (*F2*) or factor X (*F10*) also exert fibrin-independent effects, for example by directly activating specific receptors present on the surface of cancer cells and cells of the tumor microenvironment (TME), including myeloid cells/immune cells (6, 14–16). In the context of hematogenous dissemination of cancer, the formation of fibrin might also contribute to the stabilization of multicellular ecosystems (17). In the blood of breast cancer patients, Circulating Tumor Cells (CTC) can either circulate as single cells or as part of cellular clusters (18, 19). An activated coagulation cascade may aid in the stabilization of such cellular aggregates, giving CTC a greater chance of achieving successful hematogenous dissemination (17). Overall, an active coagulation on the surface of cancer cells could be a potential determinant of the ability of these cells to resist and thrive despite the attack of immune cells, and survive anticancer treatment (20, 21).

The possibility that the coagulation cascade could contribute to postsurgical recurrence has recently been proposed in studies examining blood biomarkers of coagulation in OSCC patients. Caruntu et al. (22) and Liang et al. (23) reported a negative prognostic value of elevated preoperative blood fibrinogen concentrations and platelet counts in OSCC. Intratumoral platelet microthrombi observed within OSCC were also recently reported to correlate with lymph node metastasis and higher pre-operative values of D-dimers and fibrinogen, in agreement with a possible deleterious effect of the coagulation cascade in this setting (24, 25). Interesting as these studies are, they are based on the retrospective examination of small cohorts of OSCC patients. They are also subject to the obvious criticism that the biomarkers analyzed reflect systemic inflammation and liver function as much as the procoagulant state of the tumor. To the best of our knowledge, no studies have yet examined whether the tumor procoagulant status is linked to the outcome of surgical resection and the risk of postsurgical recurrence of OSCC. The aim of the present study was to directly examine this possibility. Given the power of systems approaches in addressing clinical questions related to oral tumors (3, 4), we examined the coagulome of OSCC samples with an approach combining transcriptomic data and machine learning algorithms.

## 2 Materials and methods

### 2.1 Patient data and tumor coagulome analysis

Basic clinical, pathological and mRNA expression data (RNA SeqV2 data normalised using RNA-Seq by Expectation Maximization: RSEM) were retrieved for *n* = 321 OSCC patients from the TCGA-HNSC cohort through cBioportal (http://cbioportal.org) in November 2024. Clinical data on Disease Free Survival (DFS), Overall Survival (OS) and histological factors of local recurrence were retrieved through cBioportal. We selected OSCC with a low/intermediate risk of recurrence, as described previously (26), by excluding OSCC with nodal Extracapsular Spread (ECS) or positive surgical margins (SM) (*n* = 103 retained, 72 of which had data on DFS). The GSE65858 cohort was used for independent validation (27), with gene expression analyzed by microarray (HT12 v4 Expression BeadChips, Illumina), and DFS data available for 61 primary OSCC with low intermediate risk. In each case, we retrieved gene expression data corresponding to the “Coagulation and Complement” gene set (hsa04610) in the *Kyoto Encyclopedia of Genes and Genomes* (KEGG). From a total of *n* = 88 genes, gene expression data were available for 85 genes in TCGA. Gene expression was normalized to z scores in further analyses, to allow inter-cohort comparisons. A separate cohort with 11 matched primary and recurrent OSCC (GSE173855) with RNA-seq data (Illumina HiSeq 2000) was also analyzed (28). The basic clinical information and tumor staging data for the three cohorts with bulk transcriptomics that we analyzed in this study are presented in Supplementary Table S1.

### 2.2 Model construction based on machine learning

Six different machine-learning algorithms were used to identify the coagulation genes most linked to tumor recurrence (DFS): XGBoost (eXtreme Gradient Boosting), CoxPH (Cox Proportional Hazard), LASSO (Least absolute shrinkage and selection operator), Random Forest (RF), Support Vector Machine (SVM) and LightGBM (Light Gradient Boosting Machine), using TCGA data. In each case the training/testing process was repeated 100x, with a 70:30 split. The top 20 most important features were identified in each case, retaining the consensus features. The R and Python codes for each model are provided in the Supplementary Materials section. The features that were retained for model construction were used in a CoxPH regression model, giving a coagulation score that is the sum of the product of each feature (gene z score) with its corresponding coefficient (coagulation score = *Σβ*i * xi + intercept), where xi and *β*i are the retained features and corresponding coefficients.

### 2.3 Single-cell RNA analyses of OSCC

Single-cell RNA-seq data from 5,902 cells were obtained from GSE103322. Pre-processing and quality control of the scRNA-seq data are described in detail in Puram et al. (29). The data include 2,215 malignant cells obtained from 18 HPV negative OSCC, and 3,687 other cell types. Expression is given as TPM (transcripts per million) values.

### 2.4 Tumor microenvironment analyses, gene set enrichment analysis (GSEA)

The CIBERSORTx algorithm (https://cibersortx.stanford.edu) was used to quantify the levels of 22 cell subsets using the validated leukocyte gene signature matrix LM22 (30, 31). MCP Counter algorithm measures the abundance of eight immune and two stromal cell populations from transcriptomic data (32). Gene Set Enrichment Analysis (GSEA) was performed with the Java GSEA desktop application (https://www.gsea-msigdb.org/gsea/index.jsp) (33), using Hallmarks gene sets to compute the enrichment of specific gene sets.

### 2.5 Statistics

The association of each coagulome gene to tumor recurrence was studied by calculating the hazard ratios (HR) and 95% Confidence intervals (CI) for DFS for each gene (Cox proportional hazards regression). Kaplan–Meier analyses and the log-rank test were used to compare the DFS and OS in OSCC tumors with high/low coagulation score, divided by the median. Analyses were done with R version 3.4.2 using the packages “randomForestSRC”, “survival”, “survminer”, “ggplot2”, “gplots” (https://www.r-project.org) and Python (Anaconda/spider 5.4.3, https://www.anaconda.com/), using libraries “Pandas”, “NumPy”, “scikit-learn”, “lifelines”, “Matplotlib”, “lightgbm” and “xgboost”. Correlation analyses were carried out using R Pearson correlation.

## 3 Results

**Figure 1:**
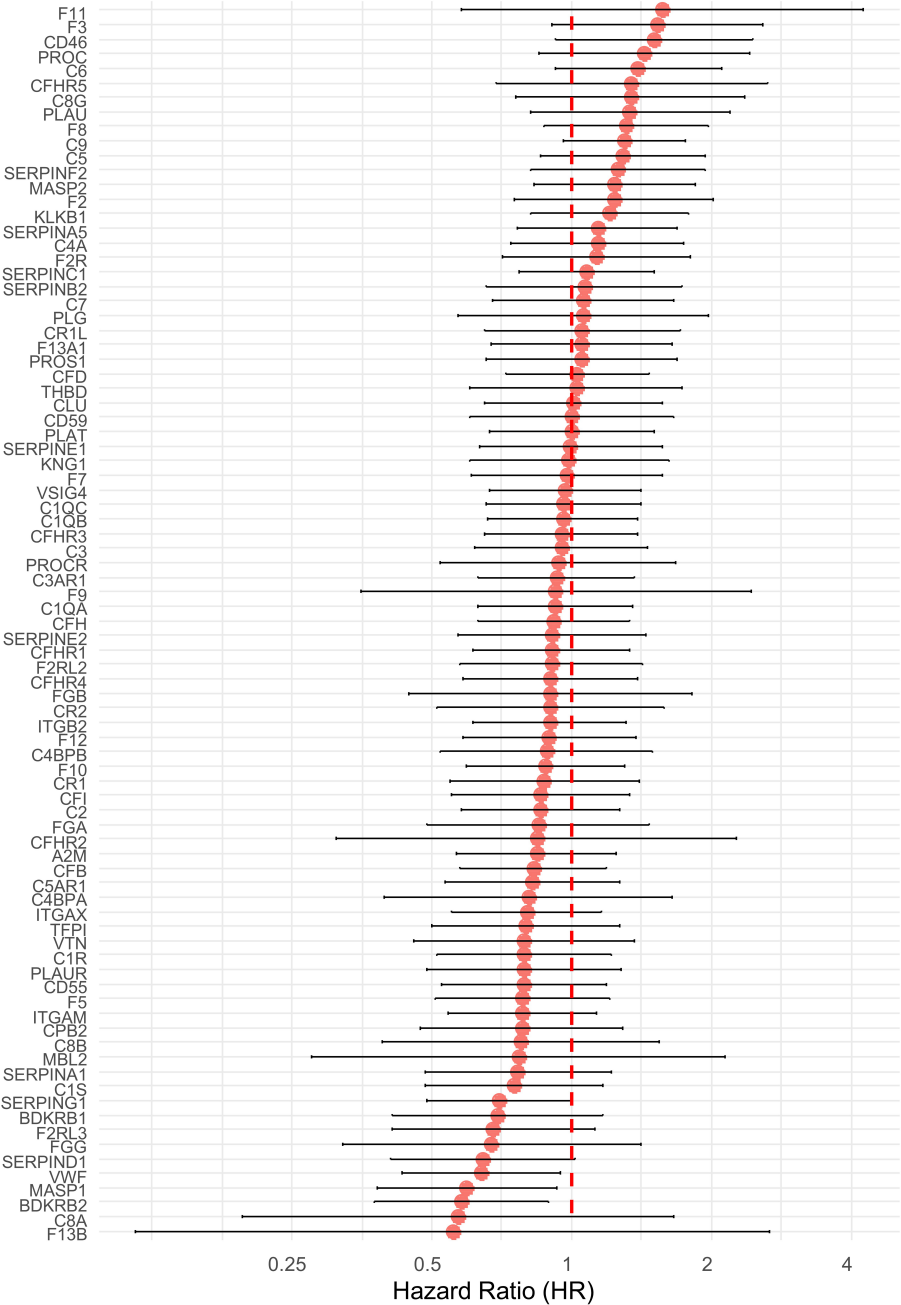
Hazard ratios (HR) and 95% confidence intervals (CI) for disease-free survival (DFS) for 85 coagulation-related genes in TCGA-OSCC patients with low/intermediate risk of recurrence. Cox proportional hazards regression was used. Hazard ratios are shown on a log2 scale. The error bars represent the limits of the 95% CI for the hazard ratio. Genes are ordered from the highest to the lowest HR. Values above 1 correspond to genes associated with an increased risk of recurrence, and values below 1 are indicative of a protective effect.

In order to address the possible link between the coagulome of OSCC and postsurgical tumor recurrence, we turned our attention to OSCC with low/intermediate risk of tumor recurrence from TCGA, *n* = 103, 72 of which had data on DFS. For each tumor, we used the z score values for the 85 genes of the KEGG gene set “Coagulation and complement” (hsa04610). For each gene, we calculated a Hazard Ratio (HR) with a 95% Confidence Interval (CI_95_) in predicting the DFS with Cox proportional hazards regression (Figure 1). This initial analysis did not identify any coagulation genes whose expression would efficiently predict tumor recurrence on its own, even though a few of the genes were close to statistical significance. Interestingly, some key regulators of CAT, such as the genes *F3* (HR = 1.53)*, F2* (HR = 1.23) and *F8* (HR = 1.31) were highly ranked in this analysis. A significant negative association with postsurgical recurrence was found with the genes *VWF* (HR = 0.64, *p* = 0.0238), *MASP1* (HR = 0.60, *p* = 0.0214) and *BDKRB2* (HR = 0.58, *p* = 0.0139).

**Figure 2:**
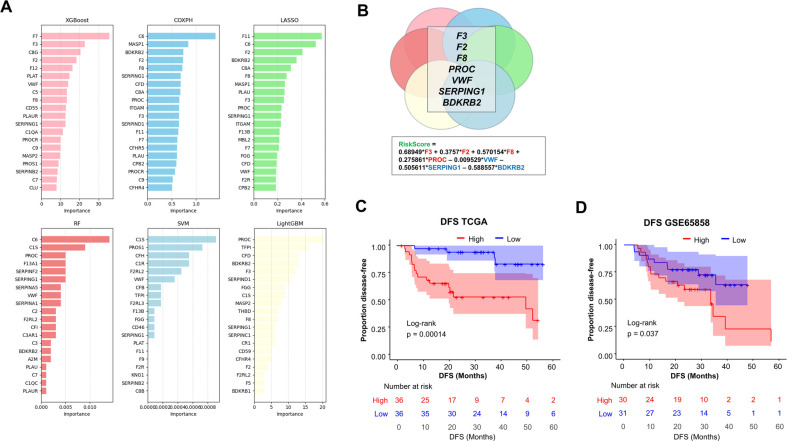
Construction of a coagulation-related gene expression model to predict post-surgical recurrence of OSCC. **(A)** Comparison of six machine-learning models for DFS prediction using 85 coagulation-related genes [XGBoost, Cox Proportional Hazards, Lasso, Support Vector Machine (SVM), Random Forest (RF), and LightGBM]. For each model, the top 20 most influential features are displayed, ordered by descending importance. **(B)** Features that were identified as important in at least four of the six models were retained for the model to obtain a coagulation score (*n* = 7 genes). **(C)** Kaplan–Meier analyses of DFS in TCGA-OSCC patients with low/intermediate risk of recurrence, stratified according to their coagulation score (by median). High score in red, low score in blue. **(D)** Validation of model performance in DFS prediction in an independent cohort (GSE65858, *n* = 61 OSCC). Note that data on *F2* were not available in GSE65858. Log-rank test was used for statistical comparisons in each case.

In order to examine the possibility that a combination of genes would better predict tumor recurrence, we applied six machine-learning algorithms: Least Absolute Shrinkage and Selection Operator (LASSO), Random Forest (RF), Support Vector Machine (SVM), XGBoost, CoxPH, and LightGBM. For each of these algorithms, we ranked the coagulation genes according to their importance in predicting tumor recurrence (Figure 2A). Interestingly, seven coagulation genes were consistently identified in the top 20 most important recurrence-predicting genes, *i.e.,* in at least four out of six machine-learning feature selection models. The corresponding seven genes (*F3*, *F2*, *F8*, *PROC*, *VWF*, *SERPING1* and *BDKRB2*) were retained in order to build a model that we tested for its ability to predict postsurgical recurrence (Figure 2B). The coagulation score values were normally distributed (Supplementary Figure S1). Interestingly, we noticed that positive coefficients applied to the key procoagulant genes *F3*, *F2*, *F8* and *PROC*, opening the possibility that the model may reflect a procoagulant tumor status (Figure 2B). A Kaplan–Meier analysis of DFS indicated that this gene expression signature predicted tumor recurrence in OSCC from TCGA. DFS was significantly lower in OSCC tumors with a high score, compared to tumors with a low score (divided by median), *p* = 0.00014 (Figure 2C). Consistently, we found that it also predicted an earlier mortality in this population [overall survival (OS), *p* = 0.012] (Supplementary Figure S2). In order to provide an independent validation, we turned our attention to a separate cohort. In the study of Wichmann et al. (27), bulk tumor transcriptomic and survival data were available for 61 low/intermediate risk OSCC. In this cohort, high values of the coagulation gene expression signature were significantly related to reduced DFS, *p* = 0.037 with log-rank test (Figure 2D). We concluded that our coagulation gene expression signature holds potential to identify OSCC at risk of postsurgical recurrence.

**Figure 3:**
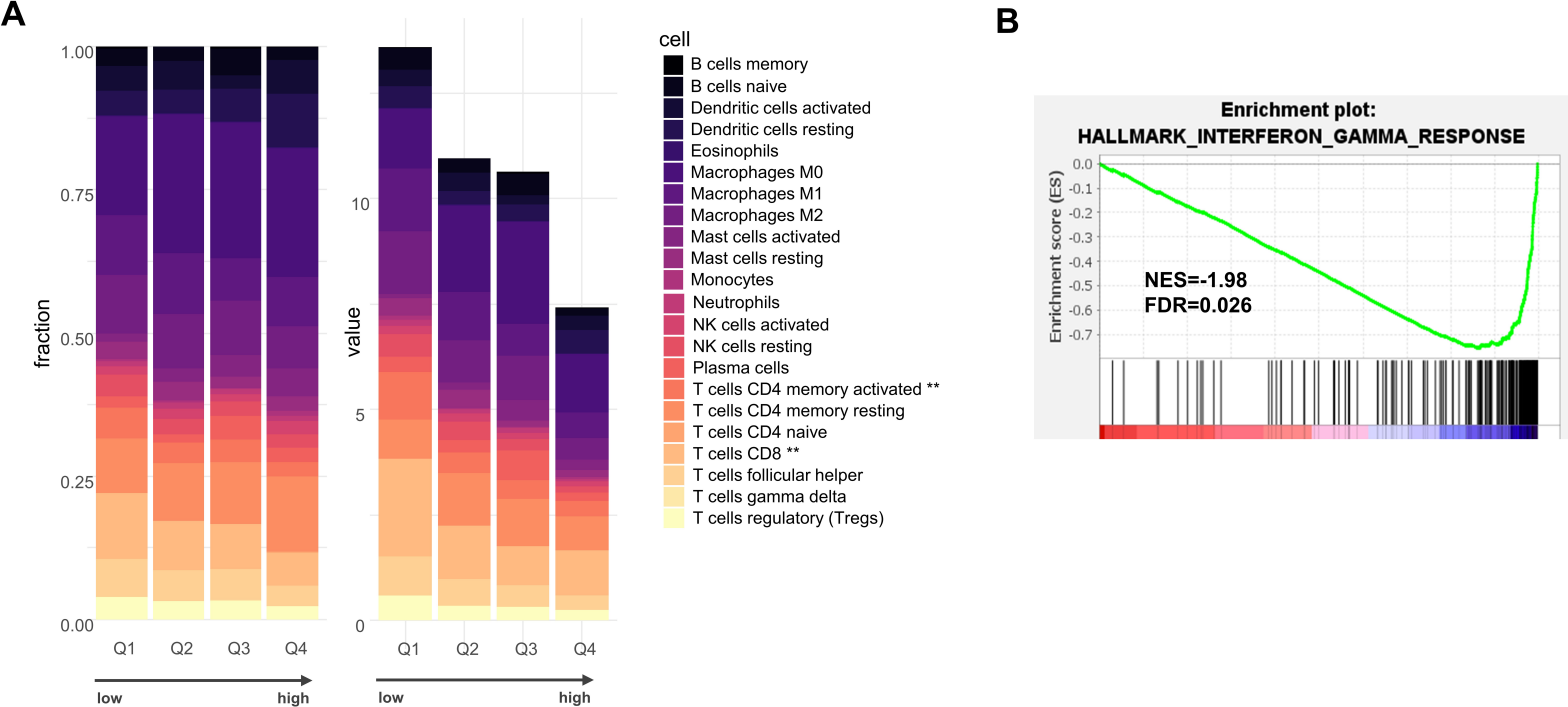
**(A)** CIBERSORTx analysis of infiltration levels of 22 different immune cell subtypes in TCGA-OSCC tumors ranked according to their coagulation score, organized into quartiles Q1–Q4, with Q1 with the lowest score and Q4 with the highest score. The results are shown as relative fractions (left, Chi2 = 1) or absolute scores (right, **p < 0.01 using Kruskal–Wallis). **(B)** Gene Set Enrichment Analysis (GSEA) on TCGA-OSCC tumors according to the score (low vs. high, by median). NES = normalized enrichment score.

**Figure 4:**
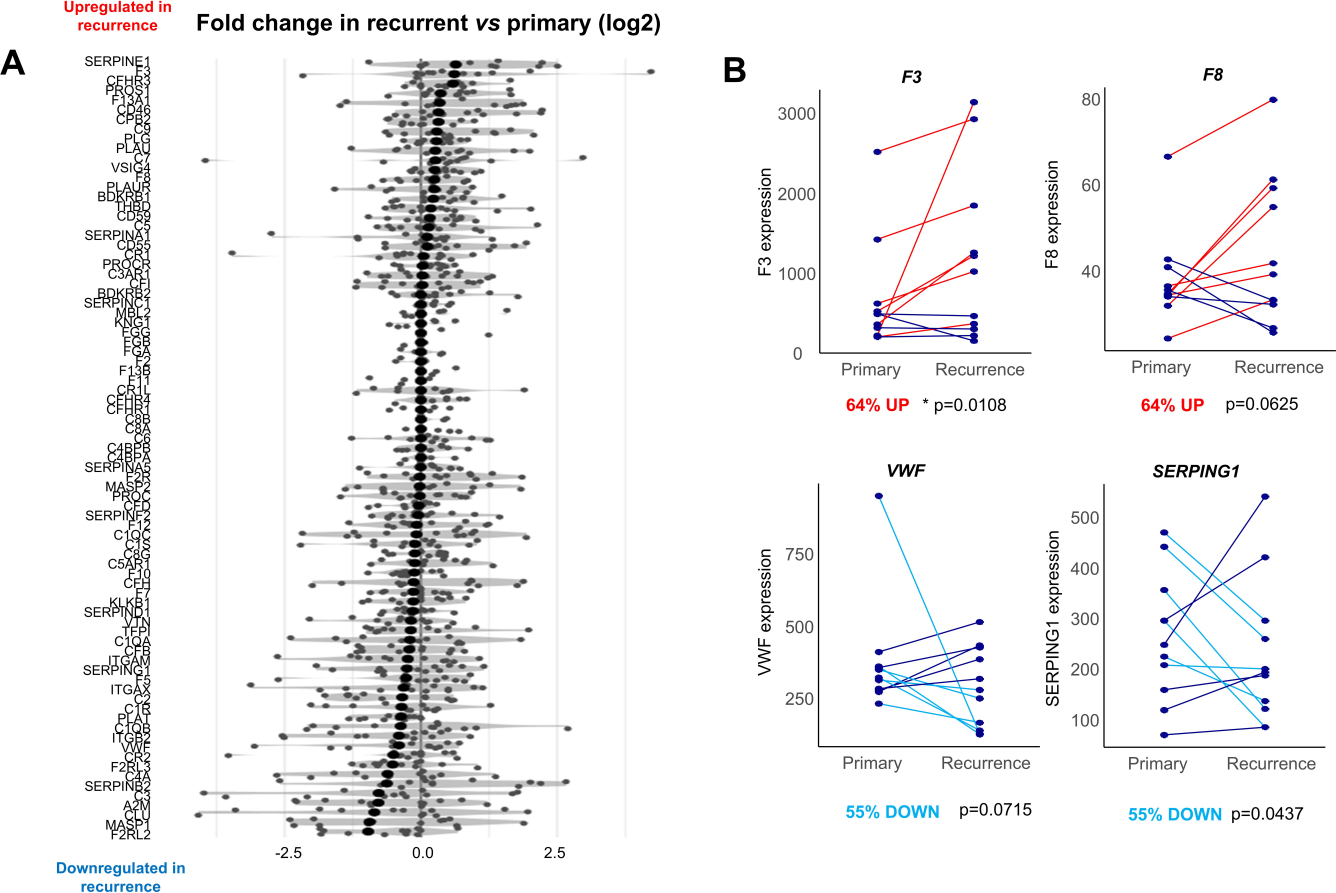
Comparison of the coagulome of matching primary vs. recurrent OSCC. **(A)** The 85 genes of the coagulome are sorted in descending order of fold-change in recurrent vs. primary tumor samples. **(B)** Direct comparison of the expression of *F3*, *F8*, *SERPING1* and *VWF* in matched primary/recurrent samples. *p* values obtained with paired Wilcoxon signed-rank test. The % of tumors with up- or down-regulation of each gene in tumor recurrence are indicated.

We explored the regulation of the seven genes that comprise our signature. Genomic analyses in TCGA-OSCC indicated that copy number alterations and somatic mutations were infrequent for these genes in OSCC (Supplementary Figure S3A). We also examined the levels of DNA methylation in TCGA-OSCC. A strong negative correlation between gene expression and DNA methylation was found for the gene *F3* (Pearson *r* = −0.60), suggesting the possible contribution of epigenetics in gene expression regulation (Supplementary Figure S3B). We next explored the contribution of the different cell types that are present in OSCC, using single-cell RNA-seq data (scRNA-seq) from the study by Puram et al. (29) (Supplementary Figure S3C). This analysis suggested the contribution of multiple cell types to the gene expression signature. Cancer cells were found to express key procoagulant genes, such as *F3*, *F2* and *PROC*. In addition, endothelial cells expressed high levels of *F8*, *VWF* and *BRDKRB2*, fibroblasts expressed *SERPING1*, and T cells and tumor macrophages expressed *F2* and *PROC*, respectively. To further analyze the pathological significance of the coagulation signature, we turned our attention to the TME of OSCC. We stratified the tumors according to the coagulation score, based on quartiles (Q1–Q4). We performed an analysis of the cellular composition of the TME with two different algorithms: CIBERSORTx (30, 31) and MCP counter (32). Digital cytometry with CIBERSORTx indicated that the absolute infiltration levels of the combined immune cell component was lower in tumors with a high coagulation score, with an almost 2-fold difference between Q1 and Q4 (Figure 3A). CD8T cells and CD4 memory activated *T* cells were the most significantly differentially represented cell types (Kruskal–Wallis, *p* = 0.0053 and *p* = 0.0070, respectively). This conclusion was independently supported with MCP counter (Supplementary Figure S4). We also performed a GSEA using Hallmarks gene sets, and observed that OSCC with low values of the coagulation score were enriched in the Hallmark “Interferon_Gamma_Response”, with a normalized enrichment score (NES) of 1.98 and *p* = 0.026 (Figure 4B). The gene *CD274*, encoding the immune checkpoint ligand PD-L1 (Programmed Death-Ligand 1) ranked highly in the corresponding leading edge analysis. We confirmed the lower expression of PD-L1 and PD1 expression in OSCC with high values of the coagulation score (Supplementary Figure S5).

Given the availability of bulk transcriptomic data for *n* = 11 matched primary/recurrent OSCC samples form a separate study (28), we next directly compared the coagulome of primary vs. post-surgical recurrence of OSCC. A direct comparison of the expression identified the genes *SERPINE1* and *F3* as the highest up-regulated coagulation genes in recurrent samples compared to matched primary OSCC (Figure 4). The corresponding genes were upregulated in 73% (*p* = 0.0036) and 64% (*p* = 0.0108) of matched recurrent compared to corresponding primary samples, respectively (paired Wilcoxon signed-rank test).

## 4 Discussion

Despite current optimal preoperative tumor staging and the availability of multimodal treatments, postsurgical recurrence remains a major problem in OSCC. A better understanding of the biological mechanisms involved in recurrence could help identify biomarkers and assist physicians in their assessment of postoperative risk (3, 4). In the present study, we examine for the first time the link between the coagulome of OSCC and the risk of postsurgical recurrence and propose a gene expression signature with a prognostic value in two independent cohorts. An overview of the genes identified as positively linked to OSCC recurrence is interesting, since it includes *F3*, *F2*, *F8* and *PROC*, which respectively encode Tissue Factor, Prothrombin, Factor VIII and Protein C, *i.e.,* four positive regulators of the coagulation cascade with an established role in CAT (7). Importantly, the gene *F3* had the highest weight in the model, opening the interesting possibility that these genes might overall reflect a local procoagulant state of OSCC. We found that OSCC with the highest values of the coagulation signature had significantly reduced infiltration levels of immune cells, especially *T* cells, an important immune cell population present in OSCC. Consistently, we observed a reduced Interferon-gamma signature and lower levels of mRNA encoding the immune checkpoints PD-L1/PD1. A direct comparison of matched primary and recurrent tumor samples separately identified some of the procoagulant genes from the signature, such as *F3*, as being significantly upregulated upon recurrence. Overall, our study highlights for the first time a possible link between the tumor coagulome and postsurgical recurrence of OSCC.

Our study has a number of limitations, such as the lack of protein analyses. Whether the variations in gene expression that we analyzed here convert to comparable variations in protein expression/tumor procoagulant activity remains to be shown. Another limitation pertains to the lack of clinical data and information regarding treatments administered to OSCC patients in the cohorts analyzed here. While perioperative thromboprophylaxis can be proposed to cancer patients after surgery, multiple anticoagulants and protocols are available and the corresponding information is not available in TCGA (34, 35). At this stage, we cannot rule out the possibility of a confounding effect. A third important limitation is inherent to the correlative nature of our study. It is at this stage not possible to conclude from our observations that the coagulation cascade directly promotes OSCC recurrence, rather than just correlates with it. Nevertheless, a strong biological rationale for such a possibility can be found in the literature. *In vitro* studies show for example how factor XIa can transduce mitogenic and pro-invasive signals in OSCC cells via specific protease-activated receptors (36). In a non-exclusive manner, the local activation of the coagulation cascade may act on cells of the TME, including immune cells. The coagulation cascade indeed exerts complex modulatory effects on anti-cancer immunity, and it has been suggested that it could contribute to tumor evasion from T cell responses (15, 16). The observation that OSCC with high values of our coagulation signature had “immune-cold” characteristics suggests that immune evasion might be a mechanism linking active coagulation to postsurgical recurrence of OSCC.

The perioperative period, *i.e.,* the short period of time—usually counted in days—around tumor resection, has been compared to a Russian roulette owing to its biological complexity and the difficult prediction of its oncological outcome (37, 38). The coagulation cascade is well-known to be active during the perioperative period, but it has until now mostly been studied as a source of postsurgical thromboembolic accidents (33, 34). Our study suggests the possibility that coagulation could also shape the oncological outcome of OSCC surgery. OSCC represent the tumor type with the highest expression of the procoagulant gene *F3*, albeit the coagulome of individual tumors appears to be extremely heterogeneous (10, 11). We argue that specific procoagulant properties of OSCC might synergize with the systemic activation of coagulation induced by the surgical procedure itself to enhance the local activation of coagulation. Given our findings, we propose that further experimental studies are required to directly address the contribution of the tumor coagulome to postsurgical recurrence and examine the possibility of personalizing OSCC surgical care based on the tumor coagulome. In the future it would be interesting to conduct prospective studies with a thorough follow-up of OSCC patients, including longitudinal protein analyses and biomarkers that reflect the cancer-procoagulant status. The HYPERCAN study (HYPERcoagulation and CANcer, NCT0262281), recruiting breast cancer patients, represents an interesting model for such studies (39). Novel approaches and strategies for a non-invasive exploration of the tumor procoagulant status might also help to validate the link between the tumor coagulome and post-surgical recurrence. The analysis of tumor microvesicles found in the saliva of OSCC patients may for example reflect the procoagulant status of OSCC (40). Systems analyses of tumor images, *i.e.,* radiomics, might also emerge as a suitable strategy for the analysis of the procoagulant status of individual human tumors, as recently suggested for gliomas (41).

## Data Availability

The original contributions presented in the study are included in the article/Supplementary Material, further inquiries can be directed to the corresponding author.
