# Supplementary material for: The tumor coagulome as a potential biological determinant of postsurgical recurrence of oral squamous cell carcinoma
Source: Front Oral Health. 2025 Feb 24;6:1554739. doi: 10.3389/froh.2025.1554739 (PMC11891189; doi:10.3389/froh.2025.1554739)
Supplement: Supplementary file 1 [file Datasheet1.docx]

Supplementary Material

1. Supplementary Figures





**Supplementary Figure 1**. Histogram of coagulation score values in n=103 TCGA-OSCC tumors with low/intermediate risk of recurrence, showing approximately normal distribution.





**Supplementary Figure 2**. Kaplan-Meier analyses using the 7-gene coagulation score for the prediction of Overall Survival (OS) in TCGA-OSCC tumors.





**Supplementary Figure 3.** Genomic, epigenetic and single-cell analyses of the seven coagulation genes in OSCC. A. An oncoprint showing the frequency of occurrence of genomic mutations or copy-number alterations (CNA). B. Correlation between gene expression and gene methylation for *F3* (Pearson r=-0.60). C. Polar plots showing single-cell expression analyses of the seven genes used in the model: *F3, F2, F8, PROC, VWF, SERPING1, BDKRB2*, in different cell types from OSCC (single-cell RNA-seq data from GSE103322). In each case, the cell type with the highest expression (median) was set as maximum.

**

**

**Supplementary Figure 4.** Microenvironment Cell Populations (MCP)-counter analysis of the infiltration levels of 8 different immune cell types as well as fibroblasts and endothelial cells in TCGA-OSCC tumors stratified according to their coagulation score by quartiles, Q1-Q4, with Q1 with the lowest scores and Q4 with the highest.

**

**

**Supplementary Figure 5.** Gene expression analysis of Immune checkpoints in TCGA-OSCC tumors stratified according to their coagulation score by quartiles, Q1-Q4, with Q1 with the lowest scores and Q4 with the highest.

***2. Supplementary Table***

**TCGA GSE65858** **GSE173855**

_______________________________________________________________________________________________

Number of patients 103 61 11

Type of samples primary primary Matching primary/recurrent

Age (median, range) 62 [19-87] 56 [35-78] 55 [47-70]

Sex M (n=64); F (n=39) M (n=48); F (n=13) M (n=9); F (n=2)

Tumor staging T1 (n=15), T2 (n=50), T1 (n=13), T2 (n=20) T1 (n=1), T2 (n=5)

T3 (n=38), T4: (n=0) T3 (n=7), T4 (n=21) T3 (n=3), T4 (n=2)

N0 (n=54), N1 (n=6) N0 (n=30), N1 (n=9) N0 (n=3), N1 (n=3)

N2 (n=25), n.a. (n=18) N2 (n=22) N2 (n=5)

Smoking history yes (n=71), no (n=29) yes (n=51), no (n=10) n.a.

n.a. (n=3)

Alcohol history yes (n=59), no (n=42) yes (n=56), no (n=5) n.a.

n.a. (n=2)

_______________________________________________________________________________________________

**Supplementary Table 1**. Basic clinical and pathological information regarding OSCC samples analyzed in this study (n.a. for not available). Note that the tumors analyzed here are those considered to be at low/intermediate risk of postsurgical recurrence, as detailed in the manuscript.

***3. Supplementary Data – Python and R codes***

### Python code for COXPH ###

import pandas as pd

import numpy as np

import matplotlib.pyplot as plt

from lifelines import CoxPHFitter

file_path = 'F:/KEGG/DFS.txt'

data = pd.read_csv(file_path, sep='\t')

X = data.iloc[:, 2:] # Gene expression features

y_status = data['DFS_status'] # Survival status (1 for event, 0 for censored)

y_time = data['DFS'] # Survival time (continuous)

X = X.fillna(0)

corr_matrix = X.corr().abs()

upper = corr_matrix.where(np.triu(np.ones(corr_matrix.shape), k=1).astype(bool))

to_drop = [column for column in upper.columns if any(upper[column] > 0.9)]

X_reduced = X.drop(columns=to_drop)

cox_data = pd.concat([X_reduced, y_status, y_time], axis=1)

cox_data.columns = list(X_reduced.columns) + ['DFS_status', 'DFS']

n_repeats = 100

feature_importances = pd.DataFrame(index=X_reduced.columns)

rng = np.random.default_rng() # New-style random generator

for i in range(n_repeats):

random_seed = rng.integers(0, 10000) # Random integer between 0 and 10000

train_size = int(0.7 * len(cox_data))

shuffled_data = cox_data.sample(frac=1, random_state=random_seed) # Use dynamic random state

train = shuffled_data.iloc[:train_size, :]

test = shuffled_data.iloc[train_size:, :]

cph = CoxPHFitter(penalizer=0.1)

cph.fit(train, duration_col='DFS', event_col='DFS_status')

feature_importances[f'Run_{i+1}'] = cph.summary['z'].abs()

feature_importances['Mean_Importance'] = feature_importances.mean(axis=1)

top_features = feature_importances['Mean_Importance'].nlargest(20)

output_path = "F:/KEGG/top_20_genes_coxph_DFS_FINAL.txt"

top_features.to_csv(output_path, sep='\t', header=['Importance'], index_label='Gene')

plt.figure(figsize=(8, 12))

top_features.sort_values().plot(kind='barh', color='skyblue', fontsize=12)

plt.title('Top 20 Features - Cox Proportional Hazards', fontsize=20)

plt.xlabel('Mean Absolute Z-Score (Importance)', fontsize=12)

plt.ylabel('Gene', fontsize=12)

plt.tight_layout()

plt.savefig("F:/KEGG/top_20_genes_coxph_plot_DFS_FINAL.png")

plt.show()

### Python code for LightGBM ###

import pandas as pd

import numpy as np

import matplotlib.pyplot as plt

from sklearn.preprocessing import StandardScaler

import lightgbm as lgb

file_path = 'F:/KEGG/DFS.txt'

data = pd.read_csv(file_path, sep='\t')

X = data.iloc[:, 2:] # Gene expression features

y_status = data['DFS_status'] # Survival status (1 for event, 0 for censored)

X = X.fillna(0)

corr_matrix = X.corr().abs()

upper = corr_matrix.where(np.triu(np.ones(corr_matrix.shape), k=1).astype(bool))

to_drop = [column for column in upper.columns if any(upper[column] > 0.9)]

X_reduced = X.drop(columns=to_drop)

scaler = StandardScaler()

X_scaled = scaler.fit_transform(X_reduced)

n_repeats = 100

feature_importances = pd.DataFrame(index=X_reduced.columns)

rng = np.random.default_rng()

for i in range(n_repeats):

random_seed = rng.integers(0, 10000)

shuffled_indices = np.arange(len(X_scaled))

rng.shuffle(shuffled_indices) # Randomly shuffle the data indices

X_shuffled = X_scaled[shuffled_indices]

y_shuffled = np.array(y_status)[shuffled_indices]

lgb_model = lgb.LGBMClassifier(

n_estimators=100,

random_state=random_seed, # Seed for LightGBM's internal randomness

subsample=0.8, # Randomly use 80% of data per iteration

colsample_bytree=0.8 # Randomly use 80% of features per tree

)

lgb_model.fit(X_shuffled, y_shuffled)

importances = pd.Series(lgb_model.feature_importances_, index=X_reduced.columns)

feature_importances[f'Run_{i+1}'] = importances

feature_importances['Mean_Importance'] = feature_importances.mean(axis=1)

lgb_top_features = feature_importances['Mean_Importance'].nlargest(20)

plt.figure(figsize=(8, 12))

lgb_top_features.sort_values().plot(kind='barh', color='blue', legend=None, fontsize=12)

plt.title('Top 20 Features - LightGBM', fontsize=16)

plt.xlabel('Mean Importance', fontsize=12)

plt.ylabel('Gene', fontsize=12)

plt.tight_layout()

plt.savefig("F:/KEGG/top_20_genes_lgbm_plot_GOOD_SEED.png")

plt.show()

output_path = "F:/KEGG/top_20_genes_lgbm_GOOD_SEED.txt"

lgb_top_features.to_csv(output_path, sep='\t', header=['Importance'], index_label='Gene')

 ### Python code for SVM ###

import pandas as pd

import numpy as np

import matplotlib.pyplot as plt

from sklearn.svm import SVC

from sklearn.model_selection import train_test_split

from sklearn.inspection import permutation_importance

file_path = 'F:/KEGG/DFS.txt'

data = pd.read_csv(file_path, sep='\t')

X = data.iloc[:, 2:] # Gene expression features

y_status = data['DFS_status'] # Survival status (1 for event, 0 for censored)

y_time = data['DFS'] # Survival time (continuous)

X = X.fillna(0)

corr_matrix = X.corr().abs()

upper = corr_matrix.where(np.triu(np.ones(corr_matrix.shape), k=1).astype(bool))

to_drop = [column for column in upper.columns if any(upper[column] > 0.9)]

X_reduced = X.drop(columns=to_drop)

svm_data = pd.concat([X_reduced, y_status, y_time], axis=1)

svm_data.columns = list(X_reduced.columns) + ['DFS_status', 'DFS']

n_repeats = 100

feature_importances = pd.DataFrame(index=X_reduced.columns)

for i in range(n_repeats):

train_size = int(0.7 * len(svm_data))

shuffled_data = svm_data.sample(frac=1, random_state=i)

train = shuffled_data.iloc[:train_size, :]

test = shuffled_data.iloc[train_size:, :]

train_X = train.drop(columns=['DFS_status', 'DFS'])

train_y = train['DFS_status']

test_X = test.drop(columns=['DFS_status', 'DFS'])

test_y = test['DFS_status']

svm_model = SVC(kernel='rbf', C=1, gamma=1 / train_X.shape[1], probability=True, random_state=i)

svm_model.fit(train_X, train_y)

results = permutation_importance(svm_model, test_X, test_y, scoring='accuracy', random_state=i)

feature_importances[f'Run_{i+1}'] = results.importances_mean

feature_importances['Mean_Importance'] = feature_importances.mean(axis=1)

top_features = feature_importances['Mean_Importance'].nlargest(20)

output_path = "F:/KEGG/top_20_genes_svm_DFS_SVM_GOOD.txt"

top_features.to_csv(output_path, sep='\t', header=['Importance'], index_label='Gene')

plt.figure(figsize=(8, 12))

top_features.sort_values().plot(kind='barh', color='skyblue', fontsize=12)

plt.title('Top 20 Features - SVM with Permutation Importance', fontsize=20)

plt.xlabel('Mean Feature Importance', fontsize=12)

plt.ylabel('Gene', fontsize=12)

plt.tight_layout()

plt.savefig("F:/KEGG/top_20_genes_svm_plot_DFS_SVM_GOOD.png")

plt.show()

### Python code for XGBoost ###

import pandas as pd

import numpy as np

import matplotlib.pyplot as plt

from sklearn.model_selection import train_test_split

import xgboost as xgb

file_path = 'F:/KEGG/DFS.txt'

data = pd.read_csv(file_path, sep='\t')

X = data.iloc[:, 2:] # Gene expression features

y_status = data['DFS_status'] # Survival status (1 for event, 0 for censored)

y_time = data['DFS'] # Survival time (continuous)

X = X.fillna(0)

corr_matrix = X.corr().abs()

upper = corr_matrix.where(np.triu(np.ones(corr_matrix.shape), k=1).astype(bool))

to_drop = [column for column in upper.columns if any(upper[column] > 0.9)]

X_reduced = X.drop(columns=to_drop)

y = pd.DataFrame({'DFS_status': y_status, 'DFS': y_time})

cox_data = pd.concat([X_reduced, y], axis=1)

n_repeats = 100

feature_importances = pd.DataFrame(index=X_reduced.columns)

rng = np.random.default_rng() # New-style random generator

for i in range(n_repeats):

random_seed = rng.integers(0, 10000) # Random integer between 0 and 10000

train_X, test_X, train_y, test_y = train_test_split(

X_reduced, y, test_size=0.3, random_state=random_seed

)

dtrain = xgb.DMatrix(train_X, label=train_y['DFS'], base_margin=train_y['DFS_status'])

dtest = xgb.DMatrix(test_X, label=test_y['DFS'], base_margin=test_y['DFS_status'])

params = {

'objective': 'survival:cox',

'eval_metric': 'cox-nloglik',

'eta': 0.1,

'max_depth': 3,

'min_child_weight': 1,

'subsample': 0.8,

'colsample_bytree': 0.8,

'seed': random_seed # Use the same seed for XGBoost

}

model = xgb.train(params, dtrain, num_boost_round=100, evals=[(dtest, 'test')], verbose_eval=False)

importance = model.get_score(importance_type='weight')

run_importances = pd.Series(importance, index=list(importance.keys())).reindex(X_reduced.columns).fillna(0)

feature_importances[f'Run_{i+1}'] = run_importances

feature_importances['Mean_Importance'] = feature_importances.mean(axis=1)

top_features = feature_importances['Mean_Importance'].nlargest(20)

output_path = "F:/KEGG/top_20_genes_xgboost_DFS_KEGG_GOOD_seed1.txt"

top_features.to_csv(output_path, sep='\t', header=['Importance'], index_label='Gene')

plt.figure(figsize=(8, 12))

top_features.sort_values().plot(kind='barh', color='skyblue', fontsize=12)

plt.title('Top 20 Features - XGBoost Survival Analysis', fontsize=20)

plt.xlabel('Mean Feature Importance', fontsize=12)

plt.ylabel('Gene', fontsize=12)

plt.tight_layout()

plt.savefig("F:/KEGG/top_20_genes_xgboost_plot_DFS_KEGG_GOOD_seed1.png")

plt.show()

### Python code for LASSO ###

import pandas as pd

import numpy as np

import matplotlib.pyplot as plt

from sklearn.model_selection import train_test_split

from lifelines import CoxPHFitter

file_path = 'F:/KEGG/DFS.txt'

data = pd.read_csv(file_path, sep='\t')

X = data.iloc[:, 2:] # Gene expression features

y_status = data['DFS_status'] # Survival status (1 for event, 0 for censored)

y_time = data['DFS'] # Survival time (continuous)

X = X.fillna(0)

corr_matrix = X.corr().abs()

upper = corr_matrix.where(np.triu(np.ones(corr_matrix.shape), k=1).astype(bool))

to_drop = [column for column in upper.columns if any(upper[column] > 0.9)]

X_reduced = X.drop(columns=to_drop)

y = pd.DataFrame({'DFS_status': y_status, 'DFS': y_time})

cox_data = pd.concat([X_reduced, y], axis=1)

n_repeats = 100

feature_coefficients = pd.DataFrame(index=X_reduced.columns)

for i in range(n_repeats):

train_data, test_data = train_test_split(cox_data, test_size=0.3, random_state=i)

cph = CoxPHFitter(penalizer=0.1) # LASSO penalization via `penalizer` parameter

cph.fit(train_data, duration_col='DFS', event_col='DFS_status')

coefficients = cph.params_.reindex(X_reduced.columns).fillna(0)

feature_coefficients[f'Run_{i+1}'] = coefficients

feature_coefficients['Mean_Coefficient'] = feature_coefficients.mean(axis=1)

top_features = feature_coefficients['Mean_Coefficient'].abs().nlargest(20)

output_path = "F:/KEGG/top_20_genes_lasso_DFS_KEGGbis.txt"

top_features.to_csv(output_path, sep='\t', header=['Coefficient'], index_label='Gene')

plt.figure(figsize=(8, 12))

top_features.sort_values().plot(kind='barh', color='skyblue', fontsize=12)

plt.title('Top 20 Features - LASSO Survival Analysis', fontsize=20)

plt.xlabel('Mean Absolute Coefficient', fontsize=12)

plt.ylabel('Gene', fontsize=12)

plt.tight_layout()

plt.savefig("F:/KEGG/top_20_genes_lasso_plot_DFS_KEGGbis.png")

plt.show()

### R code for RF ###

library(randomForestSRC)

library(dplyr)

library(ggplot2)

file_path <- "F:/KEGG/DFS.txt"

data <- read.table(file_path, sep = "\t", header = TRUE)

X <- data[, 3:ncol(data)] # Gene expression features (assumed to start at column 3)

y_status <- data$DFS_status # Survival status (1 for event, 0 for censored)

y_time <- data$DFS # Survival time (continuous)

X[is.na(X)] <- 0

surv_data <- data.frame(DFS_status = y_status, DFS = y_time, X)

rsf_model <- rfsrc(Surv(DFS, DFS_status) ~ ., data = surv_data, ntree = 500, importance = "permute")

if (!is.null(rsf_model$importance)) {

importance <- rsf_model$importance

importance_df <- data.frame(Feature = names(importance), Importance = as.numeric(importance))

top_important_features <- importance_df %>%

arrange(desc(Importance)) %>%

head(20) # Get the top 20 important features

write.table(top_important_features, "F:/top_20_genes_rsf_DFS_FINAL.txt", sep = "\t", row.names = FALSE, quote = FALSE)

plot <- ggplot(top_important_features, aes(x = reorder(Feature, Importance), y = Importance)) +

geom_bar(stat = "identity", fill = "skyblue") +

coord_flip() +

theme_minimal() +

labs(title = "Top 20 Features - Random Survival Forest",

x = "Feature",

y = "Importance") +

theme(text = element_text(size = 16))

ggsave("F:/top_20_genes_rsf_plot_DFS_FINAL.png", plot = plot, width = 12, height = 8)

print(plot)

} else {

print("No importance values were extracted from the RSF model.")

}
